# Infant Feeding Practices In a Diverse Group of Women: The Healthy Start Study

**DOI:** 10.1177/1179556518824362

**Published:** 2019-01-20

**Authors:** Jill Landsbaugh Kaar, Katherine A Sauder, Allison LB Shapiro, Anne P Starling, Brandy M Ringham, Susan L Johnson, Dana Dabelea

**Affiliations:** 1 Department of Pediatrics, School of Medicine, University of Colorado, Anschutz Medical Campus; 2 Department of Epidemiology, Colorado School of Public Health, University of Colorado, Anschutz Medical Campus; 3 Department of Biostatistics and Informatics, Colorado School of Public Health, University of Colorado, Anschutz Medical Campus

**Keywords:** nutrition, infant formula, epidemiology, breastfeeding, infant feeding

## Abstract

**Background:**

To describe infant feeding practices among a diverse group of mother-offspring pairs and identify factors associated with adherence to the American Academy of Pediatrics (AAP) recommendations.

**Methods:**

Data were analyzed from 835 mother-offspring dyads in The Healthy Start Study, an ongoing longitudinal prebirth cohort in Denver, Colorado. Maternal report of infant feeding practices was obtained at 4 to 6 months and 18 to 24 months postnatally. Practices were classified according to the following AAP recommendations: exclusive breastfeeding for first 6 months, continued breastfeeding through 12 months, and introduction of solid foods around 6 months of age. Participants who met all 3 recommendations were categorized as “adherent.” All others were categorized as “not adherent.”

**Results:**

About 77% of dyads did not adhere fully to the AAP recommendations. Women who worked ⩾35 hours/week or had a higher prepregnancy body mass index were more likely to be nonadherent. Women who were older, college educated, or had offspring with greater weight for gestational age at birth were less likely to be nonadherent.

**Conclusions:**

Most of the women in a large contemporary cohort are not adhering to AAP infant feeding recommendations. Our results highlight the specific subgroups of women who may need additional support to optimize infant feeding practices.

## Introduction

The first 12 months of life are a critical time for infants to learn about food, flavors, and eating. Eating behaviors with lifelong implications begin developing as infants transition from a milk-only diet to one that includes a variety of food textures and types.^1^ Nutrition during infancy is important for future health. The long-term benefits of exposure to breast milk during this critical time are well-documented.^2–4^ Compared with children who were exclusively formula fed, children who are breastfed have lower risk of respiratory infection, otitis media, and sudden infant death syndrome.^5^ Specific feeding approaches, including differences in formula content (ie, low versus high protein), during infancy have been linked to rapid weight gain in the first year of life.^6^ Rapid weight gain during this time may increase a child’s risk of obesity.^7^ Due to the importance of infant feeding practices during the first year of life and the potentially lifelong impact of these decisions on the health of the individual, the American Academy of Pediatrics (AAP) recommends that infants should be exclusively breastfed for the first 6 months, begin consuming solid foods around 6 months, and continue breastfeeding until at least 12 months of age.^8^

Breastfeeding rates in the United States have been increasing in recent years in accordance with the Centers for Disease Control and Prevention (CDC) Division of Nutrition, Physical Activity, and Obesity’s primary goal to improve the health of both mothers and children. In 2011, the CDC initiated national efforts to increase breastfeeding rates, including keeping mothers and babies together throughout the postpartum hospital stay, promoting maternal-infant skin-to-skin contact immediately following birth, and discouraging hospitals from providing mothers with gift bags that include infant formula. These efforts have been further supported by an increase in health insurance coverage for costs associated with breastfeeding, such as professional breastfeeding support and breast milk pumps. According to the most recent “CDC Breastfeeding Report Card,” 77% of US infants started their lives on breast milk alone.^9^ By 6 months, however, only 49% of infants were still receiving breast milk, which further declined to 27% at 12 months. Thus, despite the efforts of numerous organizations including the CDC, US Special Supplemental Nutrition Program for Women, Infants, and Children (WIC), Expanded Food and Nutrition Education Program (EFNEP), Bright Futures, etc, parents are still choosing other approaches to feeding their infants.

Prior literature examining the factors related to early infant feeding decisions is limited. In a small group of urban Hispanic mothers living in Kentucky, the most significant predictor of exclusive breastfeeding at 4 months postpartum was the presence of a positive primary intimate relationship for the mother and the mother’s self-reported breastfeeding self-efficacy score assessed at 1 month postpartum.^10^ The Generation R study reported that early introduction of solids was predicted by single-parent status, infant day care use, younger maternal age, lower educational level, increasing parity, and exclusive breastfeeding for <4 months.^11^ One potentially influential factor is maternal working status. One study of more than 1700 mothers in Hong Kong reported that ~85% of women returned to work within 10 weeks postpartum and only one-third of these women continued to breastfeed for more than 2 weeks after they started back to their jobs.^12^ This study also reported that the women most likely to continue breastfeeding after returning to work were those with greater formal education. In the United States, 64.7% of women with children under age 6 years and 58.6% of women with infants under a year old work outside the home.^13^ The Family Medical Leave Act (FMLA) policy provides up to 12 weeks of unpaid, job-protected leave per year for qualified family- and medical-related reasons, including maternity leave.^14^ However, this policy does not apply to all employers, nor all employees at any particular workplace. Thus, many women of child-bearing age in the United States do not have access to maternity leave, which could influence their infant feeding decisions.^15^

To our knowledge, there is a gap in the literature regarding adherence to such guidelines as the AAP infant feeding recommendations and further, if the same factors that predict breastfeeding also predict adherence to these combined recommendations. Therefore, we examined infant feeding practices in relation to the AAP recommendations within a diverse group of women participating in a longitudinal, prebirth cohort in Denver, Colorado.^16,17^ Our goal was to estimate the proportion of women meeting or not meeting the recommendations and identify the sociodemographic factors, including maternal working status, that are associated with adherence to the recommendations.

## Methods

### Participants

Participants in this analysis were mother-child pairs participating in the Healthy Start Study, an ongoing longitudinal study that is following participants from early pregnancy through 4 to 6 years of age. Pregnant women were recruited from the University of Colorado Hospital obstetric clinic between 2009 and 2014. Women were eligible for Healthy Start if they were ⩾16 years of age, expecting a singleton birth, were <24 weeks gestation at the time of enrollment, and had no history of serious chronic disease, prior stillbirth, or extremely preterm birth (<25 weeks gestation). A total of 1410 women were enrolled and completed research visits in early pregnancy (median 17 weeks), mid-pregnancy (median 27 weeks), and at delivery (median 1 day). Postnatal research visits occurred at 4 to 6 months (median 5 months) and 18 to 24 months (median 22 months). The study was approved by the Colorado Multiple Institutional Review Board and all participants provided written informed consent.

### Study variables

**Figure 1.**
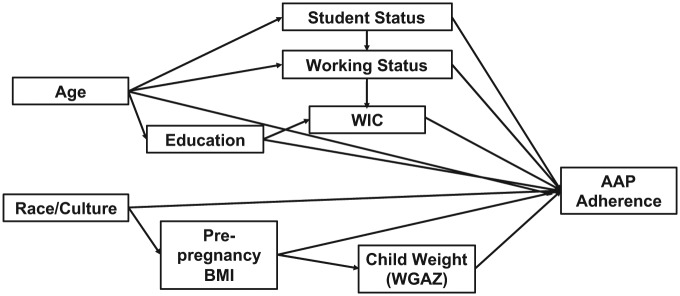
Conceptual framework for describing predictors of adherence.

The sociodemographic variables of interest and hypothesized interrelationships are depicted in Figure 1. Demographic data were collected at the research visits and through medical record abstraction. Women self-reported race/ethnicity, highest level of education completed, enrollment in WIC, student status (categorized as not a student, full-time student, part-time student), weekly working hours at 4 to 6 months (categorized as 0, 1-24, 25-35, >35 hours/week), and duration of “staying at home” (categorized <1, 2, 3, 4-5, 5-10 months postpartum, and not employed). Maternal age at delivery (years) was calculated from date of delivery and date of birth. For the analysis, maternal age was categorized as <24, 24-35, and >36 years). Maternal prepregnancy body mass index (BMI) was calculated from medical record prepregnancy weight (92%) or self-reported prepregnancy weight and measured height at the early pregnancy visit. Offspring weight-for-gestational-age *z* score was calculated from the medical record birthweight and gestational age at birth using previously published standards.^18^ Mothers’ intention to feed their child breast milk was queried at the delivery visit and a yes/no variable was created.

Infant feeding practices were assessed by maternal report at both postnatal visits. Mothers were queried on current use of breast milk, infant age when breast milk was completely stopped, use of formula, age formula was started on a daily basis, mixed feedings of both breast milk and formula, and age at which solid foods began to be consumed on a daily basis (defined as 2 or more consecutive days). Breastfeeding exclusivity through 6 months and age of introduction to solids were determined with data collected at the 4- to 6-month visit and supplemented with data from the 18- to 24-month visit when necessary. For example, for women who reported current breastfeeding at the 4- to 6-month visit, duration of breastfeeding was determined at the 18- to 24-month visit. Self-reported breastfeeding and infant feeding practices have been shown to be both valid and reliable within 3 years.^19^

### Statistical analysis

Analyses were conducted in SAS 9.4 (SAS Institute, Cary, NC, USA). Infant feeding practices were classified according to the following AAP recommendations: (1) exclusive breastfeeding for about 6 months, (2) continued breastfeeding through 12 months, and (3) introduction of solid foods around 6 months of age.^20^ Participants who met all 3 recommendations were categorized as “adherent” and all others were categorized as “not adherent.” Group differences in sociodemographic variables of interest were evaluated using *t* tests for continuous variables and the Cochran-Mantel-Haenszel test for categorical variables. Univariate logistic regression was used to estimate odds of nonadherence to the AAP recommendations. All sociodemographic variables that were significant predictors of nonadherence in univariate models were then entered into a multivariable logistic regression model. As introduction to solids recommendations vary,^8,21,22^ we conducted a sensitivity analysis in which all models were rerun using 4 months as the classification for introduction of solids. The α value ⩽.05 was considered statistically significant.

## Results

The postnatal (up to 18-24 months) research visits were completed by 1143 mother-offspring pairs as of October 14, 2016. For this analysis, 308 mother-offspring pairs were excluded due to missing demographic data (ie, working hours, access to WIC services); therefore, complete data were available on 835 dyads. There were no differences between the analytic sample (N = 835) and full cohort (N = 1410) in terms of maternal age (28.4 vs 28.1 years) and race/ethnicity (57% vs 53% non-Hispanic white) (data not shown). See Table 1 for characteristics of the analytic sample.

**Table 1.** Characteristics of cohort by adherence to the American Academy of Pediatrics infant feeding guidelines^a^.

	Analytic cohort		Adherent		Nonadherent	
	N = 835		N = 195		N = 640	
*Working status characteristics*						
Hours worked by mother at 4–6 mo postpartum						
0 hours/week (not currently employed)	319	38%	65	33%	254	40%
1–24 h/wk	129	15%	41	21%	88	14%
25–34 h/wk	82	10%	20	10%	62	10%
35+ h/wk	305	37%	69	37%	236	37%
Months of postpartum leave						
<1	37	4%	11	6%	26	4%
2	151	18%	36	18%	115	18%
3	263	32%	68	35%	195	30%
4–5	51	6%	14	7%	37	6%
5–10	14.0	2%	1	1%	13	2%
Not employed	319	38%	65	33%	254	40%
*Sociodemographic characteristics*						
Age, y						
<24	198	25%	15	8%	183	30%
24–35	528	67%	146	80%	382	62%
>36	68	9%	21	12%	47	8%
Race/ethnicity						
Hispanic	195	23%	25	13%	170	27%
Non-Hispanic white	475	57%	152	78%	323	51%
Black	109	13%	9	5%	100	16%
Other	56	7%	9	5%	47	7%
Education						
<High school degree	100	12%	6	3%	94	15%
High school degree	131	16%	10	5%	121	19%
Some college	604	72%	179	92%	425	66%
Student status (ref.: not a student)						
Not a student	720	86%	181	93%	539	84%
Full-time student	58	7%	6	3%	52	8%
Part-time student	57	7%	8	4%	49	8%
WIC services	202	24%	20	10%	182	28%
*Biological characteristics*						
Prepregnancy body mass index	25.7	6.2	24.0	4.9	26.2	6.4
Offspring weight-for-gestational-age *z* score	−0.40	0.9	−0.20	0.9	−0.46	0.9

Values are mean (SD) or No. (%).

a Mothers who met all 3 recommendations were categorized as “adherent” and all others were categorized as “not adherent.”

Overall, 77% (n = 640) of mothers did not fully adhere to the AAP infant feeding recommendations. Of these, 78% (n = 499) did not exclusively breastfeed for the first 6 months, 84% (n = 538) did not continue breastfeeding for 12 months, and 39% (n = 250) introduced solids prior to 6 months. Of the women who did not exclusively breastfeed for 6 months, 77% introduced formula in the first 3 months. Of the 265 women who did exclusively breastfeed for 6 months and continued breastfeeding for 12 months, 37 (14%) reported using formula in 6 to 12 months. Over 85% of these nonadherent mothers reported at the hospital postdelivery that they intended to feed their baby breast milk, whereas 100% of women who adhered to the AAP recommendations documented intention (data not included in subsequent analysis due to lack of variability to detect significance).

In univariate analyses (Table 2), characteristics that predicted a greater odds of nonadherence included younger age category (odds ratio [OR] = 4.66, 95% confidence interval [CI] 2.66-8.16), higher prepregnancy BMI (OR = 1.45 per 5 kg/m^2^, 95% CI 1.23-1.71), identifying as a minority race/ethnicity (Hispanic OR = 3.23, 95% CI 2.04-5.13; black OR = 5.28, 95% CI 2.60-10.73; other OR = 2.48, 95% CI 1.19-5.20), receiving WIC services (OR = 3.54, 95% CI 2.16-5.80), and being a full-time student (OR = 2.61, 95% CI 1.17-5.85). Characteristics that predicted a lower odds of nonadherence (ie, more likely to adhere) included working <24 hours/week (OR = 0.54, 95% CI 0.34-0.84), being older (OR = 0.65 per 5 years, 95% CI 0.56-0.75), having at least some college education (OR = 0.21, 95% CI 0.11-0.40), and having a larger baby at birth (OR = 0.72 per SD unit, 95% CI 0.60-0.86).

**Table 2.** Predictors of nonadherence to American Academy of Pediatrics infant feeding guidelines.

	Univariate		Multivariable	
	OR (95% CI)		OR (95% CI)	
*Working status characteristics*				
Hours worked by mother at 4–6 months postpartum				
0 h/wk (not currently employed)	Reference		Reference	
1–24 h/wk	**0.54**	**(0.34–0.84)**	1.40	(0.81–0.43)
25–34 h/wk	0.80	(0.45–1.42)	1.76	(0.93–3.35)
35+ h/wk	0.89	(0.61–1.30)	**2.09**	**(1.32**–**3.35)**
Months of postpartum leave				
<1	0.82	(0.52–1.30)		
2	0.73	(0.50–1.08)		
3	0.68	(0.35–1.33)		
4–5	3.33	(0.43–25.90)		
5–10	0.61	(0.28–1.29)		
Not employed	Reference			
*Sociodemographic characteristics*				
Age, y				
<24	**4.66**	**(2.66**–**8.16)**	1.87	(0.96–3.64)
24–35	Reference		Reference	
>36	0.86	(0.49–1.48)	0.81	(0.45–1.44)
Race/ethnicity				
Hispanic	**3.23**	**(2.04**–**5.13)**	1.50	(0.85–2.63)
Non-Hispanic white	Reference		Reference	
Black	**5.28**	**(2.60**–**10.73)**	2.03	(0.92–4.48)
Other	**2.48**	**(1.19**–**5.20)**	1.78	(0.81–3.89)
Education				
<High school degree	1.41	(0.50–3.96)	1.46	(0.47–4.58)
High school degree	Reference		Reference	
Some college or college degree	**0.21**	**(0.11**–**0.40)**	**0.35**	**(0.17**–**0.75)**
Student status				
Not a student	Reference		Reference	
Full-time student	**2.61**	**(1.17**–**5.85)**	**3.17**	**(1.08**–**9.36)**
Part-time student	2.08	(0.97–4.47)	1.41	(0.62–3.24)
Received WIC services	**3.54**	**(2.16**–**5.80)**	1.67	(0.94–2.98)
*Biological characteristics*				
Prepregnancy body mass index (per 5 kg/m^2^)	**1.45**	**(1.23**–**1.71)**	**1.30**	**(1.08**–**1.57)**
Weight-for-gestational-age *z* score at birth	**0.72**	**(0.60**–**0.86)**	**0.76**	**(0.62–** **0.93)**

Abbreviations: CI, confidence interval; OR, odds ratio.

Multivariable: multiple logistic regression was used to identify independent predictors of nonadherence to the AAP recommendations, entering all significant univariate predictors in a single model. Significant findings are in bold font.

In a multivariable analysis that included all significant univariate predictors, the sociodemographic characteristics that were associated with an increased odds of nonadherence were working ⩾35 hours/week (OR = 2.09, 95% CI 1.32-3.35), being a full-time student (OR = 3.17, 95% CI 1.08-9.36), and higher prepregnancy BMI (OR = 1.30 per 5 kg/m^2^, 95% CI 1.08-1.57). Characteristics that predicted a lower odds of nonadherence included having at least some college education (OR = 0.35, 95% CI 0.17-0.75) and having a larger baby at birth (OR = 0.76 per SD unit, 95% CI 0.62-0.93). These results were unchanged in the sensitivity analysis that classified introduction to solids at 4 months (data not shown).

## Discussion

We found that most of the women in a contemporary cohort are not following the AAP recommendations for infant feeding in the first year of life. Women were most likely to not adhere if they were working at least 35 hours/week at 4 to 6 months postpartum or had a higher prepregnancy BMI. Women were less likely to not adhere (ie, more likely to adhere) if they were older, had at least some college education, or delivered babies who were larger at birth. Our study highlights differences in infant feeding in a large, diverse cohort of women and identifies sociodemographic predictors of nonadherence that can be targeted in future efforts to optimize infant feeding.

The AAP guideline least likely to be followed is the recommendation for exclusive breastfeeding. Several previous studies have found, in univariate analyses, that women who are younger, have a lower income, are less educated, or are racial/ethnic minorities, are less likely to initiate breastfeeding, and are more likely to have a shorter breastfeeding duration.^23^ In a nationally representative sample of women from the Early Childhood Longitudinal Study Birth Cohort (ECLS-B), Ogbuanu et al^24,25^ found that non-Hispanic white, older (30+ years), more educated women with higher income status had the highest rates of predominant breastfeeding up to 3 months and any breastfeeding up to 6 months. The ECLS-B cohort also observed that the later the women returned to work, the more likely they were to initiate breastfeeding and continue with at least “some” breastfeeding beyond 6 months, with women returning at or after 13 weeks postpartum having had the highest odds of predominately breastfeeding for more than 3 months.^24^ In contrast, full-time working mothers were significantly less likely to initiate breastfeeding than mothers not employed, and full-time working mothers were also significantly less likely to breastfeed beyond 6 months.^25^ Similar findings have been documented in other countries showing maternal employment as a major predictor of introducing formula within the first 6 months.^26^ A study in India examined breastfeeding exclusivity for the first 6 months, and although women had an option of maternity leave, mothers supplemented with formula due to the “fear that the child may not take the bottle feed later” after they return to work.^27^

We also found that higher maternal prepregnancy BMI was associated with greater odds for nonadherence to feeding guidelines, independent of other sociodemographic variables, which is consistent with prior studies that have reported reduced breastfeeding initiation and duration among obese women.^19,28–30^ There are multiple reasons why obese mothers have less breastfeeding success than normal weight mothers, including but not limited to alterations in hormone regulation after delivery that affect lactation and milk secretion, increased risk of pregnancy complications (ie, cesarean delivery, preterm births), and more frequent infant latching difficulties.^31^ Obese mothers are less likely to receive the pro-breastfeeding in-hospital initiatives compared with normal weight mothers.^32^ Other studies have shown that mothers with higher prepregnancy BMI are more likely to introduce solids at an earlier age, although the factors for this behavior are not known.^33,34^ Our results suggest that obese women may need additional support throughout the first year of life to meet infant feeding recommendations.

We found in our cohort that the higher the child’s weight-for-gestational-age *z* score, the more likely the mother was to adhere to the recommendations. Previous studies have found that mothers report “low” or “insufficient” milk supply as a major reason for supplementing formula,^35–37^ which is commonly seen among babies who are bigger at birth. Mothers report that they do not feel they make enough milk to feed their baby and hence they choose to supplement with formula. Because we restricted our analysis to term births, this finding cannot be attributed to preterm offspring who often require high-calorie formula supplementation shortly after birth. We are not aware of other studies linking term infant birthweight to adherence to AAP recommendations.

In contrast to prior work,^23–25^ we did not observe a difference in infant feeding practices by maternal race/ethnicity after adjustment for other sociodemographic predictors including WIC participation, education, and prepregnancy BMI. As shown in our conceptual framework, it is possible that the relationship between race/ethnicity and infant feeding was confounded by maternal prepregnancy BMI because obesity prevalence is notably higher among racial/ethnic minorities, as well as lower education and income levels.^38^ These prior studies examined individual predictors of infant feeding practices only and did not examine all predictors simultaneously as we did in our multivariable analysis. Future work needs to consider all possible predictors in their models to ensure that we fully understand which sociodemographic variables influence infant feeding practices.

Two other variables that were significantly related to AAP adherence in univariate models but not multivariable models were student status and WIC participation. Conceptually, we think the attenuation of the association between “student” status and nonadherence from the univariate to multivariable model may be attributed to students in the analysis cohort being younger than nonstudents. Similarly, the univariate associations of WIC participation with nonadherence were also attenuated in the multivariable model. A recent study reported no association between WIC participation and breastfeeding at 3 months postpartum.^39^ In our sample, the univariate association between WIC participation and adherence may have been confounded by lower maternal education and maternal prepregnancy BMI.^37^

This study has some limitations and several strengths. Data for assessing adherence to AAP recommendations were based on the mother’s recall of her infant feeding practices at each study visit. Thus, these data are subject to reporting errors. However, evidence suggests that women can accurately report their feeding practices years later.^40^ We determined age of introduction to solids based on the age when mothers reported feeding solid foods to infants on a daily basis, which does not account for small tastes of foods that may have occurred earlier but irregularly. We also recognize that the AAP infant feeding recommendation for introducing solid foods is “around 6 months” and can be interpreted as 4 to 6 months.^8^ We performed a sensitivity analysis that classified adherence to introduction of solids based on 4 months, instead of 6 months, and the results were unchanged. Every effort to minimize reporting errors was made using the same questions at each postnatal visit and determining adherence to each recommendation based on data collected at the research visit occurring most immediate to the time period of interest. Although some work environments are more conducive to continued breastfeeding even after returning to work, we did not have data on occupation to investigate this characteristic of employment, nor did we have data on whether the maternity leave was paid or unpaid. Use of WIC services was assessed only at enrollment, and it is possible that WIC participation changed from early pregnancy to 12 months after delivery. Finally, although our cohort in Denver, CO, was diverse and representative of Colorado, our findings may not be generalizable. Strengths of the study include the prospective cohort design, a large ethnically diverse sample of infants in the United States, as well as the multiple measures of infant feeding practices over the course of the child’s first 12 months of life. In summary, a substantial proportion of mothers in this large, contemporary cohort are not adhering to the AAP infant feeding recommendations, predominately because they are not exclusively breastfeeding. Nonadherence was strongly predicted by a greater number of hours the mother worked at 4 to 6 months postpartum and by higher prepregnancy BMI. In a sensitivity analysis examining each recommendation separately, the findings for breastfeeding exclusivity and continuation remained unchanged. Of note, none of our variables were significantly associated with adherence of introduction to solids.

Health professionals working with mothers of infants need to be aware of these factors and should initiate conversations in pregnancy and the early postpartum period about potential barriers to optimal infant feeding. Given the potential role that a woman’s employment may have on her infant feeding decisions, policy changes in the workplace to support working women to breastfeed seem to be warranted. In addition to the national programs (CDC, WIC, EFNEP, etc) to promote baby-friendly hospitals and breastfeeding initiation, increased efforts are needed to address the barriers to continued breastfeeding through 6 to 12 months. Novel strategies that have worked in other countries, specifically interventions using personalized lactation consultations,^41^ should be considered.
